# 

**Published:** 1906-04-14

**Authors:** 


### Medical Appointments and Vacancies.

### APPOINTMENTS.

Hay, John, M.D., M.R.C.P., M.R.C.S., Honorary Physician to the Hospital for Consumption and Diseases of the Chest, Liverpool.

### VACANCIES.

Aberdeen (New) Poorhouse.—Resident Assistant Medical Officer.

Ayr County Hospital.—Resident House Surgeon.

Bridgwater Hospital.—House Surgeon.

Buxton, Derbyshire, Devonshire Hospital.—Assistant House Surgeon.

Canterbury, Kent and Canterbury Hospital.—House Physician.

Carlisle Non-Provident Dispensary.—Resident Medical Officer.

Carnegie Dunfermline Trustees.—Assistant Medical Officer (female).

Central London Throat and Ear Hospital, Gray's Inn Road, W.C.—Honorary Registrar.

Chester General Infirmary.—House Physician.

Committee for the Study of Special Diseases.—Research Scholarship.

Cotford, Taunton, Somerset and Bath Asylum.—Assistant Medical Officer.

Dudley, Guest Hospital.—Assistant House Surgeon.

East London Hospital for Children and Dispensary for Women, Shadwell, E.—Assistant Surgeon.

Garlands, Carlisle, Cumberland and Westmorland Asylum. Junior Assistant Medical Officer.

Gartloch Asylum, Gartcosh.—Assistant Medical Officer.

Hospital for Women, Soho Square, W.—House Physician.

Hull Royal Infirmary.—Honorary Assistant Ophthalmic Surgeon.

Ingham Infirmary and South Shields and Westoe Dispensary.—Senior House Surgeon.

Knighton Union.—District Medical Officer.

Leith Hospital.—Medical Registrar.

Liverpool Infirmary for Children.—House Surgeon.

Liverpool Royal Southern Hospital.—Junior House Surgeon.

Liverpool Stanley Hospital.—House Surgeon.

London Lock Hospital.—House Surgeon to the Female Hospital.

Maidstone, West Kent General Hospital.—House Surgeon. Also Assistant House Surgeon and Anæsthetist.

Manchester Hospital for Consumption and Diseases of the Throat and Chest.—Resident Medical Officer for the In-patient Department at Bowdon, Cheshire.

Margate, Royal Sea Bathing Hospital.—Resident Surgeon, as Junior and Senior.

Paddington Green Children's Hospital, London, W.—House Physician, also House Surgeon.

Paisley, Royal Alexandra Infirmary, Barbour Park.—House Surgeon.

Poplar Hospital for Accidents, Poplar, E.—Assistant House Surgeon.

Reigate Town Council and Rural District Council.—Medical Officer of Health.

Royal College of Surgeons of England.—Four Examiners in Anatomy and Four Examiners in Physiology.

Royal Free Hospital, Gray's Inn Road, W.C.—House Physician and Casualty House Surgeon. Also Resident House Physician and Resident House Surgeon (Female). Also Assistant to the Clinical Pathologist. Also Assistant Anæsthetist. Both females.

Royal Halifax Infirmary.—Second House Surgeon.

Royal Hospital for Diseases of the Chest, City Road, E.C.—Resident Medical Officer.

St. George's Union Infirmary, Fulham Road, S.W.—Second Assistant Medical Officer.

Shrewsbury, Salop Infirmary.—House Surgeon.

Swansea General and Eye Hospital.—House Surgeon.

Tottenham Hospital, London, N.—House Surgeon, House Physician. Casualty Officer.

Virginia Water, Surrey, Holloway Sanatorium, St. Ann's Heath.—Junior Assistant Medical Officer.

West Riding of Yorkshire.—Asssistant to County Medical Officer.

### NOTICE TO CORRESPONDENTS.

**All MSS., Letters, Books for Review, and other matters intended for the Editor should be addressed The Editor, "The Hospital," The Hospital Buildings, 28 & 29 Southampton Street, Strand, W.C.**

**The Editor cannot undertake to return rejected MS., even when accompanied by stamped directed envelope.**

### Notice to Advertisers and Subscribers.

**All Advertisements, Orders for Copies of The Hospital, and Business Communications should be addressed to THE MANAGER (Not to the Editor), The Hospital, 28 & 29 Southampton Street, Strand London, W.C.**

**The Cost of Subscription, post free, is as follows:—Per week, 3½d.; per quarter, 3s. 3d.; per half-year, 6s. 6d.; per annum, 13s. Foreign and Colonial:—Per annum, 19s. 6d., payable, in all cases, in advance.**

**NOTICE.—Vol. XXXVIII. of "The Hospital" April to September 1905), in handsome cloth binding, will shortly be on sale at the office of this Journal, price 10s. 6d. Cases for binding the Numbers contained in this Volume 2s. Index to Vol. XXXVIII., 3½d., post free.**

**The Monthly Part for April is now ready. Price 1s., by post, 1s. 3d.**

### Books for Midwives and Maternity Nurses

*Just Published.*

### SIMPLE INTRODUCTORY LESSONS IN MIDWIFERY.

By M. LOANE, Author of "Outlines of Routine in District Nursing." **1/-** net, **1/1** post free

This book is an easy introduction to the theory of Midwifery, for the use of those women who desire ultimately to pass the Central Midwives Board Examination, and who have perhaps some practical experience of midwifery, but who are so entirely ignorant of the theory, and so completely unacquainted with the language in which it is usually set forth, that they are unable to profit immediately by any ordinary courses of lectures on the subject.

The Best and Latest Handbook on Midwifery.

### A COMPLETE HANDBOOK OF MIDWIFERY.

By Dr. J. K. WATSON, Author of "A Handbook for Nurses." **6/-** net, **6/4** post free.

This Handbook has been designed to meet the requirements of the Central Midwives Board. It contains a Complete Course of Midwifery, is very fully illustrated, and is undoubtedly the best Textbook for Nurses entering for the C.M.B. Examinations, as it is only by a thorough knowledge of the subject that success can be ensured.

A Valuable Work of Reference for Practising Midwives.

### A PRACTICAL HANDBOOK OF MIDWIFERY.

By FRANCIS W. HAULTAIN, M.D. New and Revised Edition, profusely Illustrated with Original Cuts, Tables, &c.; handsomely bound in leather. **6/-** net, **6/3** post free.

### Useful Handbooks.

### HOW TO BECOME A CERTIFIED MIDWIFE.

By E. L. C. APPEL, M.B., B.S., B.Sc. Lond. Cloth, **1/-** net, **1/1** post free.

### NURSING NOTES ON MIDWIFERY AND GYNÆCOLOGY.

By Sister ROSS, Rotunda Hospital, Dublin. (Suitable size for apron pocket) Cloth, **2/-** net, **2/1** post free.

### THE MIDWIVES' POCKET BOOK (With Prefatory Chapter on the Midwives Bill).

By HONNOR MORTEN. Cloth, **1/-** post free.

### THE MOTHER'S HELP AND GUIDE TO THE DOMESTIC MANAGEMENT OF HER CHILDREN.

By P. MURRAY BRAIDWOOD, M.D. Second Edition, with Addenda, cloth, **2/-** post free.

### THE NURSING OF SICK CHILDREN.

By JAMES BURNET, M.A., M.D., M.R.C.P.Edin. Cloth, **1/-** net, **1/1** post free.

*Contains Chapters on:—*

The Feeding of Sick Children.
Baths: When and How to Give Them.
On the Management of Respiratory Cases.
Vomiting and Diarrhœa Cases.
On the Management of Rheumatic Cases.
Diseases of the Nervous System.
Paralysed and Spinal Cases.
Rickets and Scorbutus.
Skin Cases.
Diseases of the Eye, Ear, Nose, and Throat.

*Catalogue of Nursing Manuals sent post free on application.*

**The Scientific Press, Ltd.,**

**Publishers of Nursing Text-Books, Charts, and Case-Books of all Descriptions.**

**28 & 29 SOUTHAMPTON STREET, STRAND, LONDON, W.C.**

